# THE ASSOCIATION BETWEEN SMARTPHONE USE AND HEALTH BEHAVIORS AMONG OLDER ADULTS WITH DISABILITIES

**DOI:** 10.1093/geroni/igac059.2277

**Published:** 2022-12-20

**Authors:** Eunjin Yang

**Affiliations:** Yonsei University, Seoul, Seoul-t'ukpyolsi, Republic of Korea

## Abstract

People actively use smartphones to maintain and improve their health in the digital era. With this, evidence exists of the positive effect of smartphone use on the well-being of people with disabilities. This study aimed to examine the preferred sources of health information among older adults with disabilities and the association between smartphone use and healthy behaviors. This study is based on data from the 2017 national survey on persons with disabilities carried out in South Korea. A total of 4,014 participants aged 60 and above were included. Logistic regression analysis was carried out to verify the association between smartphone use and health behaviors. As the study reports, 37.7% were smartphone users and 62.3% were non-smartphone users. Among health information resources, participants identified mass media, medical institutions, and acquaintances as the first, second, and third preferred sources, regardless of smartphone use. However, a higher proportion of non-smartphone users reported having no health information available to them compared to smartphone users (5.7% vs. 1.3%, respectively). Regarding health behaviors, older adults with disabilities who use smartphones are more likely to have regular health checkups (Odd Ratio [OR], 1.51), cancer screenings (OR 1.33), dental examinations (OR 1.26), and exercise (OR 1.47), and to consume a balanced diet (OR 1.23) compared to non-smartphone users. Although these results do not prove causal relationship, smartphone use is associated with healthy behaviors in this population. Considering that healthy lifestyles are crucial to achieving active aging, resolving the disability digital divide is needed for older adults with disabilities.

